# Cisplatin-Induced APE2 Overexpression Disrupts MYH9 Function and Causes Hearing Loss

**DOI:** 10.1158/2767-9764.CRC-24-0506

**Published:** 2025-06-20

**Authors:** Qingzhu Wang, Eric E. Irons, Wanying Zhang, Fangfang Zhao, Meng-Han Chang, Esther Dai, Joelle Jeon, Hanna Hong, Rie Maeda, Minseo Kim, Kylin A. Emhoff, Mei Yin, Belinda B. Willard, Qing Y. Zheng, Richard A. Prayson, Jordan Beach, Jennifer S. Yu, Bohua Hu, Jianjun Zhao, Jianhong Lin

**Affiliations:** 1 Department of Cancer Biology, Lerner Research Institute, Cleveland Clinic, Cleveland, Ohio.; 2 Department of Medicine, University Hospitals, Case Western Reserve University, Cleveland, Ohio.; 3 Department of Otolaryngology-Head and Neck Surgery, Case Western Reserve University, Cleveland, Ohio.; 4 Image Core, Lerner Research Institute, Cleveland Clinic, Cleveland, Ohio.; 5 Proteomics Core, Lerner Research Institute, Cleveland Clinic, Cleveland, Ohio.; 6 Department of Laboratory Medicine, Robert J. Tomsich Pathology & Laboratory Medicine Institute, Cleveland Clinic, Cleveland, Ohio.; 7 Department of Cell and Molecular Physiology, Stritch School of Medicine, Loyola University Chicago, Maywood, Illinois.; 8 Department of Radiation Oncology, Taussig Cancer Institute, Cleveland Clinic, Cleveland, Ohio.; 9 School of Behavioral and Brain Sciences, University of Texas at Dallas, Dallas, Texas.

## Abstract

Cisplatin remains a cornerstone chemotherapy for many solid tumors but is limited by dose-limiting toxicities, including nephrotoxicity, peripheral neuropathy, and ototoxicity—the latter of which disproportionately affects pediatric patients and lacks effective prevention strategies. Although therapeutic approaches to mitigate cisplatin-induced toxicity are urgently needed, the underlying mechanisms driving organ-specific injury remain incompletely understood. We previously identified apurinic/apyrimidinic endonuclease (APE) 2 as a critical mediator of cisplatin-induced acute kidney injury through disruption of mitochondrial integrity. In this study, we extend these findings to cisplatin-induced hearing loss (C-HL). We demonstrate that cisplatin selectively induces APE2, but not APE1, overexpression in murine and human outer hair cells. Using an inducible, outer hair cell–specific APE2 transgenic mouse model, we show that APE2 overexpression alone is sufficient to cause high-frequency hearing loss, accompanied by hair cell loss and stereocilia disorganization visualized by electron microscopy. Mechanistically, we identified a direct interaction between APE2 and MYH9, mapped the critical MYH9-binding domains, and demonstrated that APE2 knockdown preserved mitochondrial metabolism and protected cochlear cells from cisplatin-induced apoptosis. Notably, APE2 depletion activated an ATR–p53 signaling axis, promoting nuclear p53 localization and suppressing mitochondrial apoptotic pathways. Together, these findings reveal a noncanonical, APE2-dependent mechanism driving C-HL and suggest that targeting APE2 may offer a novel therapeutic strategy to prevent cisplatin-induced ototoxicity.

**Significance:**

These results reveal an unexpected role of APE2 via its interaction with MYH9, emphasizing the therapeutic promise of targeting APE2 for preventing C-HL in patients with cancer.

## Introduction

Platinum-based compounds, including cisplatin, oxaliplatin, and carboplatin, are widely used in chemotherapy regimens for a variety of solid tumors, including bladder, cervical, breast, colorectal, lung, gastric, and head and neck cancers. Cisplatin is a mainstay in the treatment of testicular cancer, in which it achieves remarkable rates of remission. However, cisplatin treatment also comes with a high risk of off-target toxicity, including nephrotoxicity, ototoxicity, neurotoxicity, and cataract formation (1). Cisplatin-induced ototoxicity, which results in sensorineural hearing loss (SNHL) and vestibular dysfunction, is particularly prevalent in the pediatric population, in which it is observed in as many as 90% of cases and is associated with significant decrements in quality of life. There exist no consensus guidelines for the mitigation of cisplatin ototoxicity, and the molecular mechanisms underlying cisplatin’s adverse effects remain incompletely understood. Novel strategies to counteract the side effects of cisplatin can mitigate its toxicity profile, improving both the quality of life and therapeutic window for patients with cancer.

Cisplatin’s cytotoxic activity is dependent on two parallel pathways of DNA damage. Upon cellular entry, cisplatin-associated chloride anions are rapidly aquated, producing an accumulation of reactive oxygen species (ROS), with ensuing oxidation of DNA bases to produce alterations in base structure and resulting base-mispairings and subsequent errors during DNA replication. Oxidized bases can be repaired by mismatch repair and base excision repair DNA damage response systems (2). More significant, however, is the displacement of water molecules in DNA’s nitrogenous bases, resulting in cross-linking of DNA strands and formation of bulky adducts that are repaired by the nucleotide excision repair pathway (3). Although rapidly dividing tumor cells are highly susceptible to cell death triggered by catastrophic cisplatin-induced DNA damage, the susceptibility of postmitotic somatic cells to cisplatin demands an explanation evoking tissue-specific differences in drug accumulation, cellular physiology, or DNA damage response pathways.

Recent literature has highlighted the ability of classic DNA repair pathway proteins to also trigger cell death by mitochondrial translocation in response to catastrophic DNA damage (4–6). We have reported that apurinic/apyrimidinic endonuclease (APE) 2, a base excision repair pathway protein, is necessary for cisplatin-induced kidney injury by binding MYH9 to trigger mitochondrial fragmentation and death of renal proximal tubule cells (7). Here, we provide data, from a newly generated cochlear hair cell–specific *APE2* transgenic animal model, supporting a role for APE2 in cisplatin-induced ototoxicity, operating by a similar MYH9-dependent mechanism.

Pathologic mutations in MYH9 cause hearing loss, kidney dysfunction, and hematopoietic disorders in humans, clinical features also seen with cisplatin treatment, suggesting similar pathophysiologic mechanisms. Three hexameric isoforms (IIA, IIB, and IIC) with similar structures are encoded by *MYH9*, *MYH10*, and *MYH14*, respectively. MYH9 serves important roles in cell adhesion, migration, proliferation, and differentiation. Both *MYH9* knockout (KO; ref. 8) and *MYH10* KO (9) mice are embryonically lethal, indicating the essential role of nonmuscle myosin IIA (NMIIA) in early development. There is still no conditional mouse model study utilizing outer hair cell (OHC)–specific Cre, such as *Prestin-CreERT2*^*+/−*^, to investigate the role of MYH9 or MYH10 in hearing loss. Patients with *MYH9/10/14* mutations are at risk of hearing loss. The severity of SNHL seems to be primarily influenced by specific mutations in *MYH9*, although some cases have been attributed to *MYH14* and a few to *MYH10* mutations. Importantly, patients with *MYH9* mutations, specifically those harboring substitutions at the R702 residue—situated in the compact functional SH1 helix—manifest the most profound degree of SNHL (10). Conversely, individuals possessing the p.E1841K substitution in the coiled-coil region, or those with mutations located in the nonhelical tailpiece, tend to exhibit a milder degree of SNHL, even at advanced ages. Functional studies of the three isoforms in mice have elucidated both unique and redundant functions for MYH9, MYH10, and MYH14 (11, 12). *MYH9* mutations are associated with several human syndromes, now grouped as *MYH9*-related disorders that include hearing loss, kidney disease, thrombocytopenia, and cataracts (13, 14). The variable penetrance of the cochlear‐specific phenotype in patients with *MYH9*-related disorders is possibly due to the type of *MYH9* mutation, along with variable compensation by MYH10 and MYH14 (15). The significance of *MYH14* is unknown as global *MYH14 KO* in mice has no obvious phenotype (16), suggesting a potential compensatory role of MYH9 or MYH10. In some case reports, *MYH14* mutations in patients are associated with hearing loss (17) and *MYH10* mutations with microcephaly, developmental delay, hydrocephalus, cerebral and cerebellar atrophy, and hearing loss (18). In summary, the exact mechanism underlying *MYH9*-related disease is unclear. The recent identification of MYH9 as a critical regulator of mitophagy and stereocilia may underscore its role in pathogenesis of *MYH9*‐related diseases (19, 20).

In our current study, we found that upregulation of APE2 and its enrichment to mitochondria in cisplatin-exposed cochlear hair cells precedes their death. Cells and mice with diminished or absent APE2 expression are protected from cisplatin-induced hair cell damage and hearing loss. Moreover, mice with OHC-specific APE2 overexpression develop hearing loss due to destruction of OHCs. APE2 suppressed nuclear ATR–p53 DNA damage response pathways triggered by cisplatin, instead inducing its own and p53’s translocation to the mitochondria, promoting mitochondrial apoptosis. Direct interactions between APE2 and mitochondrial MYH9 provoked mitochondrial fragmentation and were associated with impaired mitochondrial functions upon cisplatin treatment. Our current studies into this mechanism in cochlear cell lines, mouse models, and human tissues demonstrate a novel mechanism active in cisplatin-induced kidney and inner ear toxicity that also mirrors the clinical manifestations of *MYH9*-related diseases. These findings underscore the role of cellular stress responses (rather than catastrophic DNA damage) in cisplatin-induced hair cell death, highlighting the prospect of employing targeted approaches to attenuate cisplatin’s side effect profile while preserving its anticancer activity.

## Materials and Methods

### Patient tissue preparation

Cochlear specimens were obtained from healthy donors and from patients with cisplatin-related hearing loss in accordance with Cleveland Clinic Foundation Institutional Review Board approval requirements and Massachusetts Eye and Ear Infirmary. Tissues from two patients with cisplatin-induced hearing loss (C-HL) and tissues from one normal donor were included in our study.

### Auditory brainstem response test

Auditory brainstem responses (ABR) were elicited using Blackman-gated tone bursts (3 ms, 29.9/seconds, alternating polarity) at 4, 8, 11.2, 16, 22.4, 32, and 40 kHz via a closed-field TDT MF-1 speaker. Subdermal needle electrodes (Rhythmlink) were placed at the vertex (positive), under the test ear (reference), and at the base of the skull (ground). Average waveforms from 1,024 presentations were generated, amplified (20×), filtered (0.3–3 kHz), digitized (25 kHz), and stored for offline analysis. For each test frequency, recording began at 80 dB Sound Pressure Level (SPL) and decreased by 10 dB steps until the ABR waveform was no longer evident. If no response was obtained at 80 dB SPL, testing was performed at a maximum level of 90 dB SPL and decreased by 5 dB steps until the ABR waveform was no longer evident. ABR thresholds were determined by visual inspection of stacked waveforms for the lowest stimulus level that yielded repeatable waves.

### Distortion product otoacoustic emission test

The distortion product otoacoustic emission (DPOAE) at 2f1-f2 was recorded in the mouse ear canal using an ER10B+ Microphone (Etymotic) with a modified pipette tip. The two DPOAE primary tones were presented at constant levels of f1 = 65 dB SPL and f2 = 55 dB SPL at 14 f2 frequencies between 4,000 and 40,000 Hz (f2/f1 = 1.25) via two TDT MF-1 speakers. Mean noise floors were calculated from six spectra surrounding the DPOAE frequency (±150 Hz). Both ABR and DPOAE tests were performed in a double-blinded manner, in which both the experimental group allocation and the test personnel were blinded during data acquisition and analysis.

### Cell culture

The murine House Ear Institute-Organ of Corti 1 (HEI-OC1) cell line was kindly provided by Professor Federico Kalinec (House Ear Institute, Los Angeles, CA, USA). HEI-OC1 cells were cultured at 33°C and 10% CO_2_ in DMEM (#11965092, Gibco) containing 10% FBS (#10437028, Gibco) with penicillin/streptomycin. Where indicated, cells were treated with cisplatin (#13119, Cayman Chemical Company), prepared freshly in sterile saline, and then diluted in culture medium to concentrations ranging from 250 to 10,000 ng/mL. Treated cells were collected for analysis at indicated times or, for cell signaling immunoblots, 6 hours after treatment.

### Apoptosis assays

Cells in culture were suspended by trypsinization, washed with culture medium, and then stained with Annexin V and propidium iodide in Annexin V Binding Buffer according to the manufacturer’s instructions (BioLegend). Cells were analyzed by flow cytometry on BD LSR II, and data were analyzed using FlowJo software (RRID: SCR_008520).

### APE2 knockdown assays

HEI-OC1 cells (RRID: CVCL_D899) were seeded into flasks and allowed to adhere in an antibiotic-free medium. Cells were then transfected with antisense synthetic oligonucleotide (ASO) gapmers: GFP ASO or APE2 ASO using lipofectamine for 6 hours. Medium was replaced, and cells were collected for further analysis 24 to 48 hours later.

### RNA analysis

After treatment, cells were resuspended in TRIzol reagent (Thermo Fisher Scientific) and frozen at −80°C. RNA was extracted under RNase-free conditions according to the manufacturer’s protocol, then quantified, and normalized. Total RNAs of 100 to 1,500 ng were used for cDNA synthesis with the TaqMan MicroRNA Reverse Transcription Kit (Applied Biosystems). Then, the cDNA products were amplified by qPCR using SYBR Green PCR Master Mix (Applied Biosystems). All transcripts were normalized to control gene expression (β-actin) and then normalized to biological control as indicated.

### Real-time cell growth assay

HEI-OC1 cells were seeded into 96-well plates at 5 to 20,000 cells/well. Cell growth was assessed using an IncuCyte ZOOM live-cell image monitoring system over 4 days (Essen BioScience, RRID: SCR_019874).

### Immunoprecipitation of APE2-binding proteins

HEI-OC1 cells were transfected with the PMEV-3FLAG-hAPE2 plasmid. The FLAG tag antibody was then used to pull down APE2-binding proteins. Anti-IgG was used as a control. Pull-down samples were run on SDS-PAGE gels. The bands from the gel were cut out, washed/destained in 50% ethanol containing 5% acetic acid, dehydrated in acetonitrile, reduced with dithiothreitol, and alkylated with iodoacetamide prior to digestion. All bands were completely digested in-gel by using trypsin 5 μL (10 ng/μL) in 50 mmol/L ammonium bicarbonate and incubating overnight at room temperature. Peptides were extracted from the polyacrylamide in two aliquots of 30 μL of 50% acetonitrile containing 5% formic acid. The extracts were combined and evaporated to <10 μL in a SpeedVac and then resuspended in 1% acetic acid to make up a final volume of ∼30 μL for LC/MS analysis.

### Mass spectrometry

The LC/MS system was an LTQ-Orbitrap Elite hybrid mass spectrometer system (Thermo Fisher Scientific) and a Dionex 15 cm × 75 μm id Acclaim PepMap C18, 2 μm, 100 Å reversed-phase capillary chromatography column. Extracts were injected in 5 μL volumes, and the peptides were eluted with an acetonitrile/0.1% formic acid gradient at a flow rate of 0.25 μL/minute introduced into the mass spectrometer source. The microelectrospray ion source was operated at 2.5 kV. The digest was analyzed using the data-dependent multitask capability of the instrument, acquiring full-scan mass spectra in the Orbitrap at a resolution of 60,000 to determine peptide molecular weights and production spectra in the ion trap to enable determination of the aa sequence in sequential scans. Data were analyzed by using all the collected Collision Induced Dissociation (CID) spectra and searching the NCBI human reference sequence database (March 2015 with 99,739 entries) with the search programs Mascot (version 2.3.0) and SEQUEST (version 2.2). The data were uploaded into Scaffold (version 4.0) for protein and peptide validation. To identify proteins, a threshold of at least five CID spectra (spectral counts) was set, and the proteins identified in APE2 pull-down/control pull-down samples at a level greater than 2.5-fold were collected by filtration and marked as APE2-binding proteins for further analysis.

### Immunofluorescence microscopy

HEI-OC1 cells were seeded in 6-well plates on sterile glass coverslips and treated with cisplatin at a concentration of 250 ng/mL for 3, 6, 9, 12, and 24 hours. The samples were washed, fixed with −20°C acetone, permeabilized with 0.1% Triton X-100, and then incubated with primary antibodies, including rabbit anti-APE2 antibody (GeneTex, catalog number GTX13691, RRID: AB_367864), human anti-MYH9 antibody (GeneTex, catalog number GTX113236, RRID: AB_2037478), mouse anti-ATP5A (Abcam, catalog number ab176569, RRID: AB_2801536), rabbit anti-p53 (Santa Cruz Biotechnology, catalog number sc-6243, RRID: AB_653753), and rabbit anti-cytochrome C (Cell Signaling Technology, catalog number 4280, RRID: AB_10695410). Then the tissues were stained with secondary antibodies: goat anti-rabbit (AF488; Thermo Fisher Scientific, catalog number A-11008, RRID: AB_143165), goat anti-mouse (AF594; Thermo Fisher Scientific, catalog number A-11005, RRID: AB_2534073), and goat anti-human (AF647, Thermo Fisher Scientific, catalog number A55749-50UL, RRID: AB_2925775). Images were captured by confocal microscopy (Leica TCS SP8). Images were analyzed using Leica LAS X software. In some instances, a set of images were taken at varying z-stack depth and compressed to create a maximum intensity projection. Brightness and contrast were uniformly altered in images taken with identical settings for visualization.

For immunofluorescence staining of cochlear cells from transgenic mice, the basal-hook and middle regions were dissected out and permeabilized with 1.0% Triton X-100 for 1 hour. Nonspecific binding of secondary antibody was blocked by incubation with 10% goat serum in PBS for 1 hour at room temperature, then samples were incubated in a diluted rabbit anti-Myo7a antibody (Thermo Fisher Scientific, catalog number PA5-37182, RRID: AB_2553946) overnight at 4°C followed by incubation for 2 hours in a diluted Alexa 488–conjugated secondary antibody (Abcam), and subsequently mounted on the slide. Cochlear whole mounts were observed by confocal microscopy (Leica TCS SP8). All images were captured by confocal microscopy at 40× magnification.

### Oxygen consumption assay

The functional activity of HEI-OC1 mitochondria was measured by using a Seahorse XFe24 Analyzer and a Seahorse XF Cell Mito Stress Test Assay (Agilent Technologies). The oxygen consumption rate (OCR) of cells was measured according to the manufacturer’s instructions. Briefly, HEI-OC1 cells were pretransfected with indicated plasmids 24 hours prior to the experiment, then resuspended, and 5 × 10^4^ cells seeded into 24-well plates and allowed to attach overnight, while being treated with cisplatin at 1 μg/mL for 24 hours. Culture medium was removed and replaced with dye and buffer-free XF DMEM (Agilent Technologies), then cells subjected to OCR detection in the presence of oligomycin (1 μmol/L), carbonyl cyanide p-trifluoromethoxyphenylhydrazone (2 μmol/L), and a combination of rotenone (0.5 μmol/L) and antimycin A (0.5 μmol/L). Basal OCR = OCR before the injection of oligomycin. ATP synthesis–linked OCR (ATP-linked) = basal OCR − OCR following oligomycin injection. Maximum OCR = OCR following the injection of carbonyl cyanide p-trifluoromethoxyphenylhydrazone. Reserve = maximum respiration − basal OCR. Proton leak-linked OCR = uncoupled OCR following oligomycin − nonmitochondrial OCR following injection of rotenone and antimycin A. Nonmitochondrial OCR = OCR following the injection of rotenone and antimycin A. Significant outliers were removed prior to analysis.

### IHC

Formalin-fixed, paraffin-embedded tissue sections were deparaffinized and then incubated with a rabbit anti-APE2 polyclonal antibody (Bioss, catalog number bs-6587R, RRID: AB_11072407) and a rabbit anti-MYH9 antibody (GeneTex, catalog number GTX113236, RRID: AB_2037478) at 4°C overnight. After incubation with horseradish peroxidase (HRP)–conjugated goat anti-rabbit secondary antibody, the signal was detected using a DAB Substrate kit (Abcam, ab64238) according to the manufacturer’s instructions. Images were obtained using a phase-contrast microscope (Leica DM2000 LED) equipped with a digital camera (Leica DMC2900).

### Immunoblotting

Total cellular protein samples were isolated by RIPA buffer with protease and phosphatase inhibitors (Thermo Fisher Scientific). Where indicated, mitochondrial and cytosolic proteins were isolated using Mitochondria Isolation Kit (Thermo Fisher Scientific), per the manufacturer’s instructions. Proteins were resolved on 4% to 12% Bis-Tris polyacrylamide gels (Thermo Fisher Scientific), and transferred onto polyvinylidene difluoride membranes (Millipore). Membranes were blocked with 5% nonfat milk and incubated overnight with a rabbit anti-APE2 polyclonal antibody (Bioss, catalog number bs-6587R, RRID: AB_11072407), rabbit anti-APE1 polyclonal antibody (Novus Biologicals, catalog number NB100-116SS, RRID: AB_10701622), rabbit anti–α-tubulin (Cell Signaling Technology, catalog number 2144, RRID: AB_2210548), mouse anti-CHK1 (Cell Signaling Technology, catalog number 37010, RRID: AB_3662851), rabbit anti–CHK1-S317(Cell Signaling Technology, catalog number 2344, RRID: AB_331488), rabbit anti-Bax (Cell Signaling Technology, catalog number 2772, RRID: AB_10695870), mouse anti-ATP5A (Abcam, catalog number ab176569, RRID: AB_2801536), rabbit anti–pp53-S15 (Cell Signaling Technology, catalog number 9284, RRID: AB_331464), rabbit anti-p53 (Santa Cruz Biotechnology, catalog number sc-6243, RRID: AB_653753), rabbit anti-GAPDH (Cell Signaling Technology, catalog number 5014, RRID: AB_10693448), rabbit anti-ATR (Cell Signaling Technology, catalog number 2790, RRID: AB_2227860), rabbit anti–pATR-T1989 (GeneTex, catalog number GTX128145, RRID: AB_2687562), rabbit anti-ATM (Cell Signaling Technology, catalog number 2873, RRID: AB_2062659), rabbit anti–pATM-S1981 (Cell Signaling Technology, catalog number 5883, RRID: AB_10835213), mouse anti-PCNA (Cell Signaling Technology, catalog number 2586, RRID: AB_2160343), and rabbit anti–caspase 9 (Cell Signaling Technology, catalog number 9502, RRID: AB_2068621) overnight at 4°C. Membranes were then washed and incubated with an HRP-linked anti–rabbit IgG secondary antibody (Cell Signaling Technology, catalog number 7074, RRID: AB_2099233) or HRP-linked anti–mouse IgG secondary antibody (Cell Signaling Technology, catalog number 7076, RRID: AB_330924). Detection of chemiluminescence was carried out using a SuperSignal West Femto Maximum Sensitivity Substrate kit (Thermo Fisher Scientific).

### Scanning electron microscopy analysis

Cochleae were rapidly dissected from the cranial bone of the mouse, one animal at a time, to shorten the time between death and fixation (typically 2 minutes) at RT. Then, 500 μL of fixative, containing 4% paraformaldehyde and 2.5% glutaraldehyde in 0.1 mol/L sodium cacodylate buffer, was gently perfused through the open oval and round window, exiting through a hole made in the apical turn of the cochlea. Tissues were then postfixed overnight at 4°C on a rotating platform, rinsed 3 times with distilled water, decalcified in 10% EDTA in 100 mmol/L Tris pH 7.4 for 1 hour, and then rinsed twice again. The cochlea was dissected and postfixed in 1% osmium tetroxide for 2 hours at room temperature. The tissues were then dehydrated with serial ethanol washings from 50% to absolute ethanol, critical point dried, mounted on support stubs with carbon tabs, and sputter coated with platinum. Imaging was carried out using a ZEISS Sigma VP scanning electron microscope (RRID: SCR_023279), operating at 15 kV.

### Transmission electron microscopy analysis

Mouse cochleae were submerged in electron microscopy grade 2.5% glutaraldehyde and 4% paraformaldehyde in 0.2 mol/L sodium cacodylate buffer (pH 7.4) at 4°C immediately after collection and fixed at 4°C overnight. After washing three times for 5 minutes in sodium cacodylate buffer (0.2 mol/L, pH 7.3), cochlear fragments were fixed in 1% aqueous osmium tetroxide for 60 minutes at 4°C, then washed twice for 5 minutes with sodium cacodylate buffer and rinsed once with maleate buffer (pH 5.1, 5 minutes). After the cochlear tissues were stained with 1% uranyl acetate in maleate buffer for 60 minutes, the samples were then washed three times for 5 minutes with maleate buffer and then dehydrated with ascending grades of ethanol, and finally embedded in Epon resin (Electron Microscopy Sciences). Ultrathin sections (85 nm) were cut by means of an EM UC7 Ultramicrotome (Leica Microsystems), then successively stained with uranyl acetate and lead citrate, and examined with a transmission electron microscopy (TEM) instrument at 80 kV (Tecnai G2 SpiritBT, FEI. RRID: SCR_023279).

### Statistical analysis

A two-tailed Student *t* test was performed using Prism software (version 9.0) to compare independent pairs of groups. *P* ≤ 0.05 was statistically significant.

### Data availability

The data generated in this study are available within the article and its supplementary data files. Additional raw data and materials related to this study, including the *APE2* transgenic mouse line, *APE2* and *MYH9* plasmids, and *APE2* ASOs, are available from the corresponding author upon reasonable request and completion of a material transfer agreement.

## Results

### APE2 is enriched in mitochondria and binds to MYH9 after cisplatin treatment

**Figure 1:**
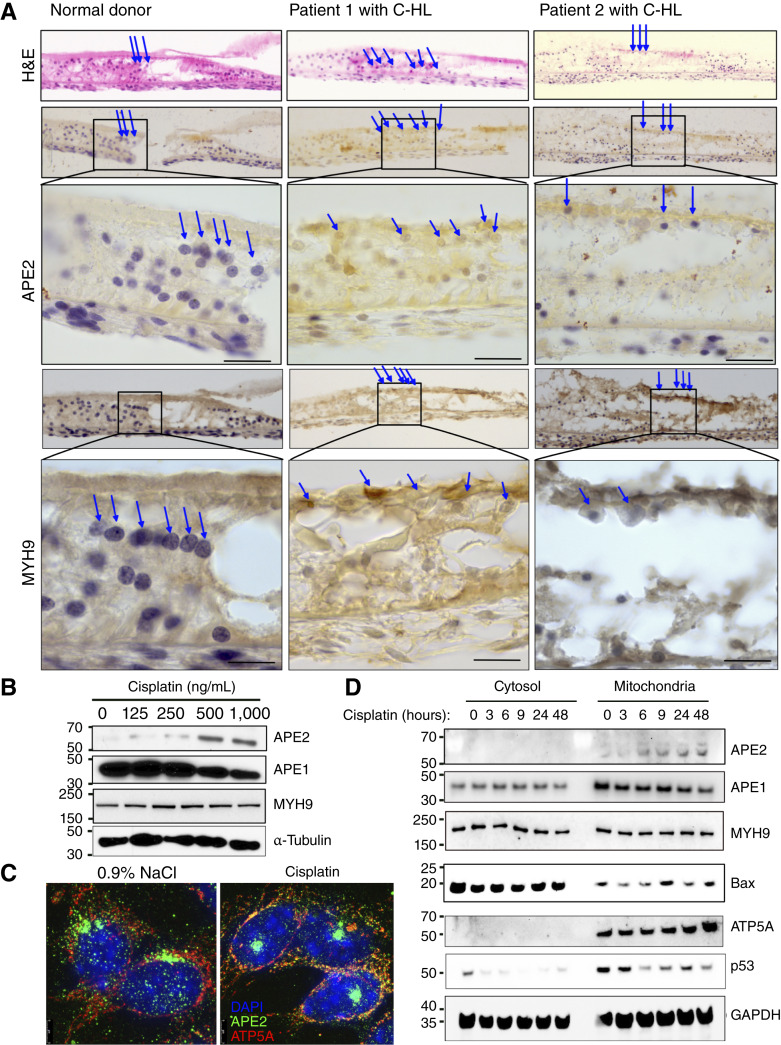
Cisplatin treatment results in APE2 upregulation and mitochondrial dysmorphology in cochlear hair cells. **A,** IHC analysis of APE2 and MYH9 expression in cochlear tissues from patients with C-HL (*n* = 2) and normal donors (*n* = 1). OHCs are highlighted using blue arrow heads. Scale bar, 50 μm. **B,** Immunoblot analysis of APE1, APE2, and MYH9 expression in HEI-OC1 cells after 24 hours of cisplatin treatment at indicated concentrations. **C,** Z-stack compressed images of HEI-OC1 cells treated with 1,000 ng/mL cisplatin for 6 hours at indicated concentrations, showing 4',6-diamidino-2-phenylindole (DAPI) (blue), APE2 (green), and ATP5A/mitochondria (red). **D,** Immunoblot of subcellular cytosolic and mitochondrial protein fractions of HEI-OC1 cells after treatment with 1,000 ng/mL cisplatin for indicated times. ATP5A mitochondrial marker is shown. The experiments shown in **B**–**D** were performed independently 3 times (*n* = 3) with consistent results. Representative data from one experiment are presented. H&E, hematoxylin and eosin.

Cisplatin-associated SNHL is attributed to the rapid and prolonged accumulation of cisplatin within the cochlear stria vascularis, which is associated with cytotoxicity in OHCs (21–23). By IHC analysis, we observed the characteristic damage to cochlear OHCs of patients who developed cisplatin ototoxicity, as reported in the literature. In addition, we observed a significant accumulation of APE2 in the cytosol of OHCs and increased expression of MYH9 granules (Fig. 1A). Analysis of DNA repair pathway protein expression in cisplatin-treated HEI-OC1 cells also revealed that APE2 was highly upregulated by cisplatin exposure, in contrast to APE1 (Fig. 1B), but not in carboplatin- and oxaliplatin-treated HEI-OC1 cells (Supplementary Fig. S2). Carboplatin and oxaliplatin have very low or rare ototoxicity in clinical use, especially compared with cisplatin, which is highly ototoxic, primarily due to their distinct chemical structures lacking chloride ligands and reduced uptake by cochlear cells via copper transporter 2 and organic cation transporter 2 (24). Interestingly, APE2 also rapidly colocalized with mitochondria, as seen in z-stack maximum intensity projection images 6 hours after cisplatin treatment (Fig. 1C). Of note, APE1 has a nuclear localization signal, and APE2 has a mitochondrial localization signal and is critical in repair of mitochondrial DNA (mtDNA) damage (25, 26). We confirmed that APE2 is present within the mitochondria as soon as 6 hours after cisplatin treatment and consistently remains upregulated 48 hours after treatment, followed by an accumulation of BAX and downregulation of p53 in mitochondria (Fig. 1D). APE1 is present in mitochondria before cisplatin treatment and downregulated in the mitochondria after cisplatin treatment (Fig. 1D). Because the ability of cisplatin to induce nuclear DNA damage *per se* is not sufficient to explain its toxic effects on normal, postmitotic tissues, we hypothesized that APE2 binding of MYH9 may be responsible for cytotoxicity in OHCs, as we previously reported in the kidney (7).

### Conditional expression of APE2 in OHCs in APE2 transgenic mice

**Figure 2:**
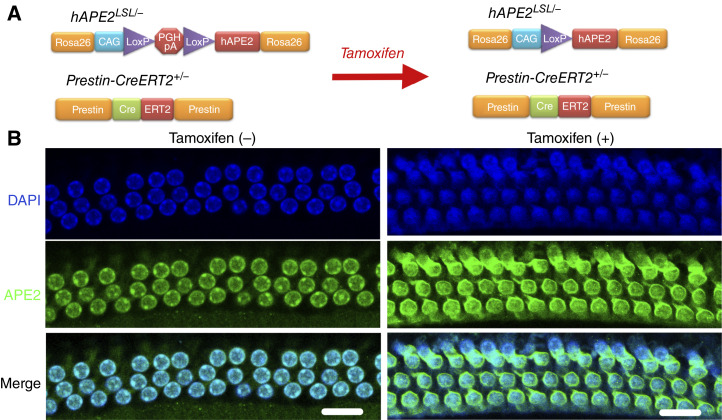
APE2 conditional transgenic mouse model. **A,** Schematic diagram of the *hAPE2*^*LSL/LSL*^ conditional transgenic mouse model. **B,** Representative images of APE2 immunofluorescence staining from OHCs of *Prestin-CreERT2*^*+/−*^*hAPE2*^*LSL/−*^ mice 7 days after tamoxifen injection compared with control mice without tamoxifen injection (*n* = 3) indicating successful Cre-induced LoxP recombination and subsequent hAPE2 overexpression in OHCs. Scale bar, 50 μm.

To determine whether C-HL develops in mice as a consequence of cisplatin-mediated effects via APE2 overexpression, we generated *APE2* conditional transgenic mice (Fig. 2A). To mimic the effect of cisplatin in OHCs, we crossed h*APE2*^*LSL/−*^ mice with *Prestin-CreERT2*^*+/−*^ mice (27). *Prestin-CreERT2* mice exhibit Cre recombinase activity specifically in OHCs upon tamoxifen induction, with no detectable activity in inner hair cells (IHC). This specificity aligns with the endogenous expression pattern of prestin, which is predominantly found in OHCs and not in IHCs. The targeted Cre activity in OHCs has been confirmed through experiments in which tamoxifen-induced Cre recombination resulted exclusively in reporter expression within OHCs, with no activity observed in IHCs. In our *Prestin-CreERT2*^*+/−*^*hAPE2*^*LSL/−*^ mice, Cre-induced expression of human *APE2* is controlled by the CAG promoter, a well-established strategy for generating transgenic mice with high expression of APE2 only in OHCs (Fig. 2B) after tamoxifen administration (28) but not in IHCs (Supplementary Fig. S3).

### APE2 transgenic mice develop C-HL–like auditory dysfunction after APE2 expression is induced

**Figure 3:**
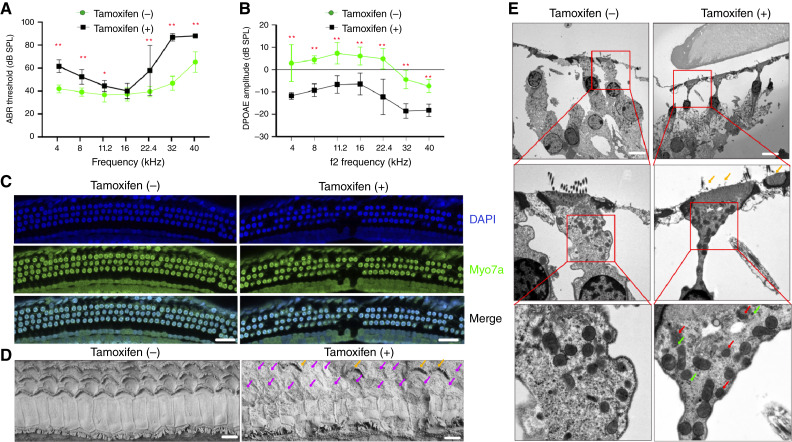
Clinical features of C-HL–like disease in APE2 transgenic mice. **A,** ABR thresholds, (**B**) DPOAE amplitudes, and (**C**) immunofluorescence staining (scale bar, 50 μm). **D,** Scanning electron microscopy (scale bar, 10 μm) in *Prestin-ERT2Cre*^*+/−*^*hAPE2*^*LSL/−*^ mice (*n* = 6) before and after injection with tamoxifen to induce APE2 overexpression. In the tamoxifen (−) group, OHCs exhibit a well-organized, intact structure, with preserved stereocilia and uniform surface morphology. In contrast, the tamoxifen (+) group shows widespread OHC degeneration, characterized by disorganized cell arrangement and significant OHC loss, as indicated by pink arrows. The damaged regions exhibit surface collapse and stereocilia disintegration (orange arrows), suggesting progressive OHC degeneration. **E,** TEM study of the mitochondria and the stereocilia of OHCs from 8-week-old* hAPE2*^*LSL/−*^*Prestin-CreERT2*^*+/−*^ mice (*n* = 3). Tamoxifen was injected in 3-week-old mice (scale bar, 10 μm). In the tamoxifen (−) group, mitochondria appear intact, exhibiting a uniform shape and electron density, indicative of normal mitochondrial function. The cytoplasmic texture remains uninterrupted, with a lack of autophagic vesicles, suggesting no active mitophagy. In contrast, the tamoxifen (+) group shows increased mitochondrial fragmentation, characterized by small, irregularly shaped mitochondria (red arrows), a hallmark of mitophagy. Additionally, the presence of electron-dense autophagic vesicles near mitochondria suggests the formation of mitophagosomes (green arrows), further supporting active mitochondrial degradation in response to APE2 overexpression. Loss of stereocilia (orange arrows), with remaining structures appearing shortened and disorganized, indicative of sensory dysfunction. *, *P* < 0.05; **, *P* < 0.01.

We then monitored and evaluated the *Prestin-CreERT2*^*+/−*^*hAPE2*^*LSL/−*^ transgenic mice for the development of hearing loss. Strikingly, *Prestin-CreERT2*^*+/−*^*hAPE2*^*LSL/−*^ mice developed irreversible hearing loss, as confirmed by ABR and DPOAE tests (Fig. 3A and B). Morphologic analysis of cochlear tissues revealed dramatic loss of Myo7a+ OHCs at the basal region of the cochlea in tamoxifen-injected hAPE2-overexpressed *Prestin-CreERT2*^*+/−*^*hAPE2*^*LSL/−*^ mice (Fig. 3C). Using scanning electron microscopy and TEM, we also observed C-HL–like pathologic features in *Prestin-CreERT2*^*+/−*^*hAPE2*^*LSL/−*^ mice, including OHC loss (Fig. 3D) and OHC apoptosis and mitophagy (Fig. 3E). Notably, TEM analysis identified mitochondrial fragmentation, autophagic vesicles, and mitochondrial matrix degradation, suggesting that APE2 overexpression triggers mitophagy as part of OHC degeneration. These findings parallel those observed in cisplatin-induced OHC damage, reinforcing that APE2 overexpression directly contributes to mitochondrial dysfunction and mitophagy-driven OHC loss. The APE2-induced hearing loss model provides a powerful tool for dissecting the mechanistic role of APE2 in C-HL, particularly its impact on mtDNA damage, mitophagy, and OHC survival.

### APE2 binds to E853 to A922 of the MYH9 tail domain

**Figure 4:**
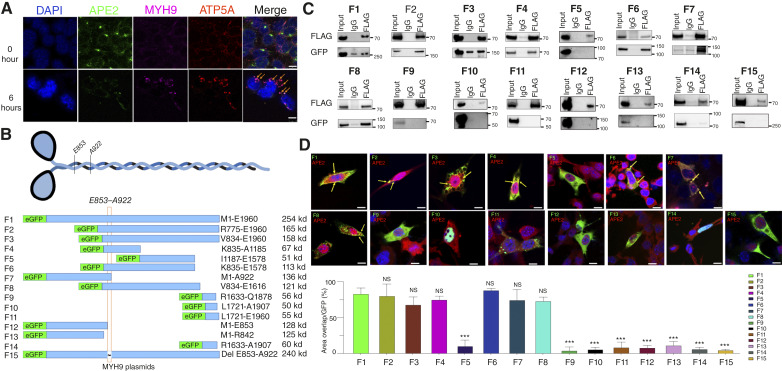
APE2 binds to the MYH9 E853–A922 domain. **A,** Confocal images of HEI-OC1 cells after treatment with 250 ng/mL cisplatin at indicated time points, with DAPI (blue), APE2 (green), ATP5A (mitochondria, red), and MYH9 (magenta). Areas of condensed mitochondria with APE2–MYH9 colocalization are indicated with orange arrowheads. The experiments shown were performed independently 3 times (*n* = 3) with consistent results. Representative data from one experiment are presented. Scale bar, 10 μm. **B,** MYH9 protein domains and the schematic diagram of enhanced Green Fluorescent Protein (GFP)-MYH9 constructs (F1–F14) used in the studies. **C,** Immunoprecipitation and (**D**) immunofluorescence of HEI-OC1 cells cotransfected with FLAG-APE2 and different GFP-MYH9 constructs (F1–F14) with quantification of the latter. Scale bar, 10 μm. The fluorescence microscopy images (top) show the localization of APE2 (red) and the MYH9-GFP constructs (green), with DAPI staining (blue) marking the nuclei. Yellow arrows indicate colocalization points. The bar graph (bottom) quantifies the percentage of APE2 colocalization relative to GFP expression for each MYH9 fragment construct (F1–F15). From the graph, it appears that constructs F1–F4 and F6–F8 have high colocalization with APE2, whereas F5 and F9–F15 show little to no colocalization. NS, no significant difference compared with F1; ***, *P* < 0.001 vs. F1.

To explore the mechanism underlying APE2-mediated hearing loss, we performed an APE2 pull-down assay followed by LC/MS proteomic analysis using FLAG-APE2, overexpressed in the HEI-OC1 mouse cochlear hair cell line, and found MYH9 and MYH10 to be among the most abundant precipitated proteins, both also specifically binding to APE2 (Table 1). To further explore the subcellular localization pattern of APE2 and MYH9 in cochlear cells during cisplatin treatment, OC-1 cells were treated with cisplatin and subjected to immunofluorescence analysis. ATP5A was used as a mitochondrial marker. As shown in Fig. 4A, in HEI-OC1 cells, APE2 was localized in both the nucleus and the mitochondria. When the cells were treated with cisplatin, both APE2 and MYH9 were colocalized in mitochondria.

**Table 1:** LS/MS analyses of APE2 pull-down proteins

Gene	Molecular weight	IgG	FLAG-APE2	APE2/IgG
*Myh9*	226	409	1,279	3.13
*Plec*	534	126	509	4.04
*Myh10*	229	75	232	3.09
*Iqgap1*	189	67	219	3.27
*Flna*	281	41	211	5.15
*Nes*	207	35	151	4.31
*Flnb*	278	16	90	5.63
*Ptbp1*	56	18	78	4.33
*Tjp1*	195	23	78	3.39
*Eef1a1*	50	24	78	3.25

We also studied the potential binding site of MYH9 on APE2 in OHCs. A full-length hAPE2-bearing 3 FLAG tags was used for the MYH9 binding experiment. The human MYH9 protein is 1,960 aa long and consists of a motor domain (head, ∼1–800 aa) for ATP and actin binding, a neck domain (∼801–900 aa) with IQ motifs, a coiled-coil rod domain (∼901–1,600 aa) for dimerization, and a tail domain (∼1,601–1,960 aa) involved in protein interactions and subcellular localization. To determine the precise binding site of MYH9 with APE2, we utilized 14 enhanced Green Fluorescence Protein (eGFP)-labeled footprint constructs of different fragment of MYH9 (Fig. 4B). We performed parallel coimmunoprecipitation and immunofluorescence microscopy on doubly transfected cells to identify the critical regions mediating APE2–MYH9 interactions. Our results revealed a strong GFP pull-down signal in F1, F2, F3, F4, F6, F7, and F8 in both coimmunoprecipitation (Fig. 4C) and immunofluorescence microscopy staining (Fig. 4D). Given the common overlapping domains among these fragments, we propose that APE2 most likely binds to the MYH9 in the connection of neck domain with coiled-coil/rod domain between E853 and A922, which is adjacent and distal to the motor domain. To further validate our hypothesis that APE2 interacts with MYH9 within the E853–A922 region, we generated a MYH9 deletion construct (*MYH9 ΔE853–A922*), designated as F15, and performed coimmunoprecipitation and immunofluorescence microscopy assays. Our findings demonstrated that APE2 failed to bind to the MYH9 ΔE853–A922 mutant in these assays (Fig. 4C and D), supporting our hypothesis that the MYH9 E853–A922 region is essential for the APE2–MYH9 interaction. Importantly, patients with MYH9 mutations specifically at the R702 residue—situated in the compact functional SH1 helix of the distal head domain—manifest the most profound degree of SNHL (29). Our observations provide a potential mechanistic explanation for hearing loss induced by cisplatin via the formation of APE2–MYH9 complexes, resulting eventually in cell death.

### APE2 knockdown mitigates cisplatin-induced apoptosis *in vitro*

**Figure 5:**
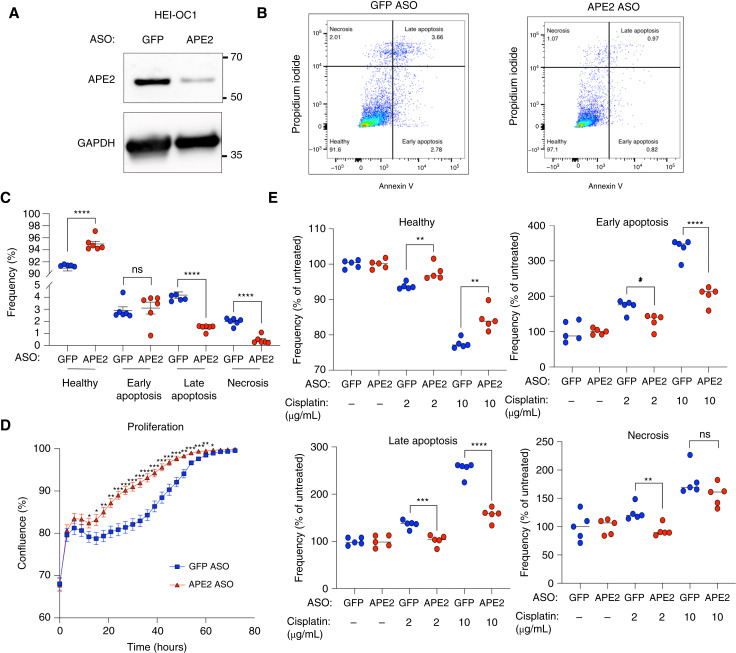
APE2 knockdown mitigates cisplatin-induced apoptosis *in vitro.***A,** Knockdown of APE2 by transfection of APE2 ASO and GFP ASO as control ASO gapmers in HEI-OC1 cells. **B,** Flow cytometry dot plots showing Annexin V and propidium iodide staining 24 hours after transfection. The experiments shown were performed independently 3 times (*n* = 3) with consistent results. Representative data from one experiment are presented. **C,** Frequency of healthy (double negatives), early apoptotic (annexin V+/PI−), late apoptotic (annexin V+/PI+), and necrotic (annexin V−/PI+) HEI-OC1 cells 24 hours after transfection. **D,** Confluence of cultures in 96-well plates seeded with 24 hours–transfected HEI-OC1 cells over 72 hours. **E,** Frequencies, normalized to respective saline controls, of healthy, early apoptotic, late apoptotic, and necrotic cells among transfected HEI-OC1 cells treated with cisplatin at indicated concentrations for 24 hours. ns, not significant; *, *P* < 0.05; **, *P* < 0.01; ***, *P* < 0.001; ****, *P* < 0.0001.

Our data thus far demonstrate that APE2 is upregulated in cochlear hair cells in response to cisplatin treatment, upregulation of APE2 is sufficient to trigger mitochondrial fragmentation and OHC cytotoxicity *in vivo*, and a major binding partner of APE2 is the mitochondrial protein MYH9. Next, we sought to understand whether loss of *APE2* will be protective against cisplatin-induced ototoxicity. To test this, we performed knockdown studies in HEI-OC1 cells using gapmer ASO constructs against APE2 mRNA (Fig. 5A). Even prior to any further treatment, we observed that APE2 knockdown decreased the proportion of early apoptotic, late apoptotic, and necrotic cells *in vitro* (Fig. 5B and C). APE2 knockdown cells also reached confluence more quickly in standard culture conditions, as measured by live-cell image monitoring (Fig. 5D). When exposed to cisplatin, APE2 knockdown protected cells from apoptotic cell death at both moderate and high doses (Fig. 5E).

### APE2 ASO confers mitochondrial protection against cisplatin injury

**Figure 6:**
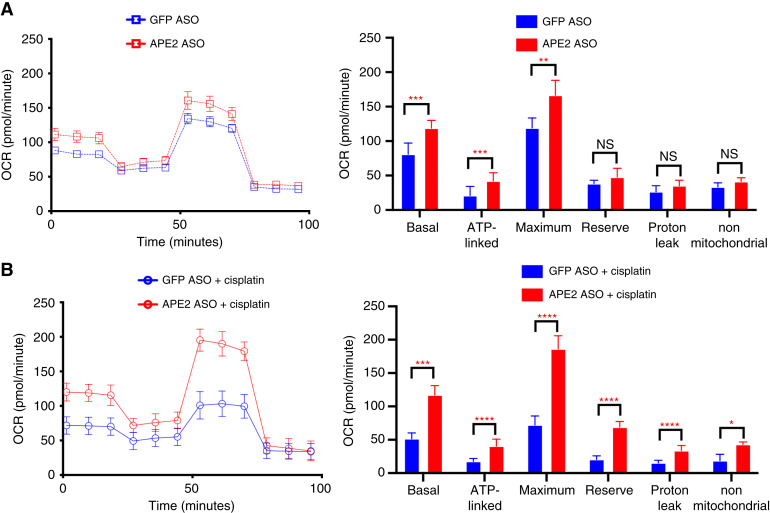
APE2 ASO restores mitochondrial respiration impaired by cisplatin treatment. **A,** Seahorse XF analysis of the mitochondrial OCR in cells transfected with control GFP ASO or APE2 ASO. Key parameters measured include basal respiration, ATP-linked respiration, maximal respiration, reserve capacity, proton leak, and nonmitochondrial respiration. **B,** Seahorse XF analysis of the OCR in cells treated with cisplatin following transfection with GFP ASO or APE2 ASO. Data represent mean ± SEM. Statistical analysis was performed using the unpaired two-tailed Student *t* test; NS, not significant; *, *P* < 0.05; **, *P* < 0.01; ***, *P* < 0.001; ****, *P* < 0.0001..

To examine the role of APE2 in regulating mitochondrial function, we conducted Seahorse XF analysis in cells transfected with GFP ASO or APE2 ASO. Under basal conditions, APE2 ASO significantly increased basal respiration, ATP-linked respiration, and maximal respiration compared with GFP ASO controls, whereas reserve capacity, proton leak, and nonmitochondrial respiration remained unchanged (Fig. 6A). These results suggest that APE2 depletion enhances mitochondrial activity without inducing overt mitochondrial stress. Following cisplatin treatment, GFP ASO–transfected cells exhibited a pronounced reduction in all major respiratory parameters, consistent with mitochondrial dysfunction. Notably, APE2 ASO–transfected cells maintained significantly higher basal, ATP-linked, maximal, reserve, proton leak, and nonmitochondrial respiration upon cisplatin exposure compared with GFP ASO–transfected cells, indicating that APE2 depletion improved mitochondrial efficiency. Together, these findings suggest that APE2 knockdown primes mitochondria for enhanced function and confers protection against cisplatin-induced mitochondrial injury.

### APE2 knockdown prevents mitochondrial translocation of p53 and preserves mitochondrial integrity in cisplatin-treated HEI-OC1 cells

**Figure 7:**
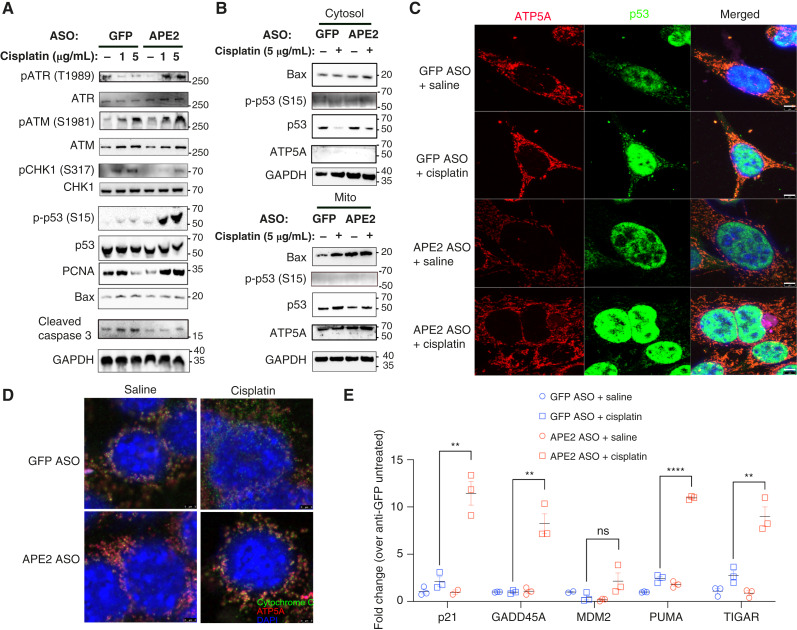
APE2 inhibits p53 signaling and provokes apoptosis. HEI-OC1 cells were transfected with indicated ASO 24 hours prior to further treatments. **A,** Cells were treated with 1 μg/mL or 5 μg/mL cisplatin for 6 hours prior to collection of total cellular protein for immunoblot analysis. **B,** Cells were treated with 5 μg/mL cisplatin for 6 hours prior to isolation of cytosolic and mitochondrial protein fractions for immunoblot analysis. Confocal microscopy analysis of HEI-OC1 cells treated with 1,000 ng/mL cisplatin for 24 hours, showing (**C**) DAPI (blue), p53 (green), and ATP5A/mitochondria (red) colocalization (scale bar, 5 μm), and (**D**) DAPI (blue), cytochrome C (green), and ATP5A/mitochondria (red) colocalization (scale bar, 3 μm). **E,** RT-qPCR analysis of expression of selected p53-associated genes in cells treated with 1,000 ng/mL cisplatin for 24 hours, normalized to β-actin and shown as fold change compared with untreated, anti-GFP transfected cells. The experiments shown were performed independently 3 times (*n* = 3) with consistent results. Representative data from one experiment are presented. ns, not significant; *, *P* < 0.05; **, *P* < 0.01; ***, *P* < 0.001; ****, *P* < 0.0001. Scale bars, 10 μm.

Given APE2’s role in DNA damage repair, we next investigated whether targeting APE2 could prevent cisplatin-induced mitochondrial dysfunction and apoptosis in cochlear HEI-OC1 cells. Specifically, we explored the impact of APE2 knockdown on DNA damage signaling and mitochondrial apoptotic pathways in the context of cisplatin exposure. Upon cisplatin treatment, control (GFP ASO–transfected) cells exhibited activation of canonical DNA damage response signaling, characterized by phosphorylation of CHK1. In contrast, APE2 knockdown cells showed a distinct phosphorylation pattern, with increased ATR activation and robust phosphorylation of p53 at Ser15 (Fig. 7A). Notably, APE2 knockdown reduced cisplatin-induced caspase-3 cleavage (Fig. 7A), suggesting decreased apoptotic activity. Although cisplatin promoted mitochondrial translocation of Bax in both control and APE2 knockdown cells, this process appeared independent of APE2 status (Fig. 7B). To further evaluate the impact of APE2 knockdown on mitochondrial integrity, we performed confocal microscopy to assess both p53 localization and mitochondrial morphology. In GFP ASO–transfected cells, cisplatin treatment triggered robust p53 translocation to mitochondria, accompanied by a fragmented and swollen mitochondrial appearance, consistent with mitochondrial injury (Fig. 7C). In contrast, APE2 ASO–transfected cells maintained largely nuclear localization of p53 following cisplatin exposure, with minimal colocalization with the mitochondrial marker ATP5A. Importantly, mitochondria in APE2 ASO + cisplatin cells exhibited a more elongated and reticulated network, resembling healthy mitochondrial morphology, in stark contrast to the fragmented phenotype observed in GFP ASO + cisplatin–treated cells. These findings indicate that APE2 knockdown preserves both mitochondrial structure and function during cisplatin-induced stress, likely through limiting p53 mitochondrial translocation and subsequent apoptotic signaling. Given that S15-phosphorylated p53 is typically retained in the nucleus, and mitochondrial p53 facilitates apoptotic signaling through Bax interactions, we examined p53 localization in subcellular compartments. Consistent with this model, S15-phosphorylated p53 was detected exclusively in the nuclear fraction, with little to no presence in cytosolic or mitochondrial compartments. These findings suggest that APE2 knockdown restricts p53 to the nucleus, potentially by promoting its S15 phosphorylation, thereby limiting its proapoptotic mitochondrial functions. Supporting this notion, cisplatin-induced cytochrome c release from mitochondria—a hallmark of mitochondrial outer membrane permeabilization—was substantially reduced in APE2 knockdown cells, indicating preserved mitochondrial membrane integrity (Fig. 7D). Finally, we examined the transcriptional consequences of nuclear p53 retention. APE2 knockdown in cisplatin-treated cells led to pronounced upregulation of multiple p53 transcriptional targets involved in cell-cycle arrest and stress response, including p21, GADD45A, PUMA, and TIGAR (Fig. 7E). Together, these findings support a model in which APE2 knockdown prevents mitochondrial translocation of p53, preserves mitochondrial morphology and integrity, and shifts p53 activity toward nuclear transcriptional responses rather than mitochondrial apoptosis (Fig. 7F).

## Discussion

Despite decades of investigation into the mechanisms of cisplatin-induced ototoxicity, there still remains significant uncertainty about the molecular pathways that trigger hair cell death in response to chemotherapeutic and other ototoxic drugs. Upon entry and aquation, cisplatin upregulates ROS production, cross-links DNA and proteins, and triggers a number of cytotoxic pathways. Much of cisplatin’s ototoxicity is thought to center on ROS-mediated disruption of mitochondrial membrane potential and oxidative phosphorylation, triggering apoptosis (30). The findings presented herein demonstrate a previously unknown role for APE2, a DNA repair enzyme, as a trigger of mitochondrial disruption and cell death in hair cells during cisplatin treatment. We show that upregulation of APE2 is sufficient and necessary for cisplatin-induced hair cell damage using a combination of *in vitro* and *in vivo* models.

Our study further extends the previous observation that APE2 interacts with nonmuscle myosin proteins such as MYH9, MYH10, and MYH14 (7). This interaction, which we demonstrated in cisplatin-treated renal proximal tubular cells, unifies the shared adverse effect profile of a number of drugs that cause both nephrotoxicity and ototoxicity with *MYH9*-related genetic diseases, which manifest with thrombocytopenia, deafness, and renal failure (31). In this study, we observed MYH9 to be predominantly colocalized with mitochondria, consistent with a previous electron microscopy study documenting MYH9 to be within mitochondrial inner membrane folds in cochlear hair cells (32). Other studies have reported that MYH9 is critical for mitochondrial fission and mtDNA partitioning, highlighting its underappreciated role in mitochondrial health (33, 34). We herein demonstrate that the APE2–MYH9 interaction is mediated by specific interdomain interactions between the APE2 and the MYH9 coiled-coil domain, suggesting a role for APE2 in modifying MYH9 functions. Given the role of APE2 in provoking mitochondrial fragmentation, we hypothesize that its mitochondrial translocation and subsequent binding to MYH9 compromises adaptive mitochondrial fission in response to cisplatin-mediated mitochondrial damage (Supplementary Fig. S4).

Between APE1 and APE2, it is already appreciated that APE2 has the unique function of repairing mtDNA, which is particularly susceptible to ROS-mediated damage. Although we did not directly analyze DNA damage, we find that loss of APE2 strongly activates ATR, but not ATM phosphorylation. A previous publication by Willis and colleagues (35) reported an integral function for APE2 in recruiting ATR to sites of ssDNA damage during oxidative stress. Our data are consistent with this as robust ATR phosphorylation in APE2 knockdown cells surprisingly did not trigger Chk1 phosphorylation. In line with literature that phosphorylation of serine 15 is executed by major DNA damage response pathway kinases ATM, ATR, and DNA-PK, we found that insufficient APE2 expression triggered phosphorylation of p53 at S15, with robust downstream transcriptional activation of canonical target genes (36). The relationship between APE2 and p53 has been previously observed in B cells, in which APE2-deficient mice experience a contraction of both bone marrow pre-B cells and splenic germinal center B cells, which can be reversed by concomitant deletion of p53 (37, 38). Importantly, p53’s transcription-independent cell death pathway, enforced by its translocation to the mitochondria and disruption of mitochondrial membrane potential by activating proapoptotic Bcl-2 family proteins (Bcl-XL, Bax, Bak, and Bcl-2), occurs concurrently with its other functions and yet is also sufficient to trigger apoptosis independently (39–41).

Despite the high incidence of ototoxicity after cisplatin treatment, there is still a lack of consensus about how to prevent, monitor for, and treat this condition (42). PEDMARK (sodium thiosulfate) was approved by the FDA on September 20, 2022, for the prevention of cisplatin-induced ototoxicity in pediatric patients (ages 1 month and older) with nonmetastatic solid tumors (43). Sodium thiosulfate protects against cisplatin-induced oxidative damage in the cochlea by detoxifying reactive platinum species, thereby reducing the risk of permanent hearing loss. However, due to concerns that sodium thiosulfate may reduce cisplatin’s antitumor efficacy, its approval is limited to pediatric patients with nonmetastatic tumors to ensure that the protective benefits outweigh potential risks to cancer treatment outcomes. For most cancers, the standard approach to prevent cisplatin-related toxicity is to administer low doses of cisplatin in combination with full intravenous hydration prior to and after cisplatin administration. Yet 30% to 50% of patients still develop C-HL due to high retention time of cisplatin in the cochlear region (23). Alternative options include the use of less ototoxic platinum chemotherapeutics (carboplatin and oxaliplatin), but they are less effective than cisplatin for certain cancers (44) and are more hepatotoxic. A large array of investigational agents exists, including those targeting ROS and reactive nitrogen species (RNS), apoptosis, G protein–coupled receptors, HSPs, and inflammation (45–54). Given its involvement in both cisplatin-mediated kidney and cochlear damage, APE2 may represent an attractive novel molecular target for small-molecule, RNA-dependent, or gene therapy approaches. In particular, our success in using ASOs in the current study and the recent development of small-molecule inhibitors of APE2 demonstrate the imminent clinical potential of this approach (55).

## Supplementary Material

Figure S1 Figure S1

Figure S2 Figure S2

Figure S3 Figure S3

Figure S4 Figure S4

Supplementary methods Supplementary methods

## References

[1] Rybak LP , Mukherjea D, Jajoo S, Ramkumar V. Cisplatin ototoxicity and protection: clinical and experimental studies. Tohoku J Exp Med 2009;219:177–86. 19851045 10.1620/tjem.219.177 PMC2927105

[2] Rocha CRR , Silva MM, Quinet A, Cabral-Neto JB, Menck CFM. DNA repair pathways and cisplatin resistance: an intimate relationship. Clinics (Sao Paulo) 2018;73:e478s. 30208165 10.6061/clinics/2018/e478s PMC6113849

[3] Schärer OD . Nucleotide excision repair in eukaryotes. Cold Spring Harb Perspect Biol 2013;5:a012609. 24086042 10.1101/cshperspect.a012609 PMC3783044

[4] Kharbanda S , Saxena S, Yoshida K, Pandey P, Kaneki M, Wang Q, . Translocation of SAPK/JNK to mitochondria and interaction with Bcl-x(L) in response to DNA damage. J Biol Chem 2000;275:322–7. 10617621 10.1074/jbc.275.1.322

[5] Erster S , Mihara M, Kim RH, Petrenko O, Moll UM. In vivo mitochondrial p53 translocation triggers a rapid first wave of cell death in response to DNA damage that can precede p53 target gene activation. Mol Cell Biol 2004;24:6728–41. 15254240 10.1128/MCB.24.15.6728-6741.2004 PMC444865

[6] Ngok-Ngam P , Watcharasit P, Thiantanawat A, Satayavivad J. Pharmacological inhibition of GSK3 attenuates DNA damage-induced apoptosis via reduction of p53 mitochondrial translocation and Bax oligomerization in neuroblastoma SH-SY5Y cells. Cell Mol Biol Lett 2013;18:58–74. 23161404 10.2478/s11658-012-0039-y PMC6275584

[7] Hu Y , Yang C, Amorim T, Maqbool M, Lin J, Li C, . Cisplatin-mediated upregulation of APE2 binding to MYH9 provokes mitochondrial fragmentation and acute kidney injury. Cancer Res 2021;81:713–23. 33288657 10.1158/0008-5472.CAN-20-1010 PMC7869671

[8] Mhatre AN , Li Y, Bhatia N, Wang KH, Atkin G, Lalwani AK. Generation and characterization of mice with Myh9 deficiency. Neuromolecular Med 2007;9:205–15. 17914179 10.1007/s12017-007-8008-8

[9] Ma X , Takeda K, Singh A, Yu ZX, Zerfas P, Blount A, . Conditional ablation of nonmuscle myosin II-B delineates heart defects in adult mice. Circ Res 2009;105:1102–9. 19815823 10.1161/CIRCRESAHA.109.200303 PMC2792753

[10] Sekine T , Konno M, Sasaki S, Moritani S, Miura T, Wong WS, . Patients with Epstein-Fechtner syndromes owing to MYH9 R702 mutations develop progressive proteinuric renal disease. Kidney Int 2010;78:207–14. 20200500 10.1038/ki.2010.21

[11] Wang A , Ma X, Conti MA, Liu C, Kawamoto S, Adelstein RS. Nonmuscle myosin II isoform and domain specificity during early mouse development. Proc Natl Acad Sci U S A 2010;107:14645–50. 20679233 10.1073/pnas.1004023107 PMC2930417

[12] Zhang Y , Conti MA, Malide D, Dong F, Wang A, Shmist YA, . Mouse models of MYH9-related disease: mutations in nonmuscle myosin II-A. Blood 2012;119:238–50. 21908426 10.1182/blood-2011-06-358853 PMC3251230

[13] Heath KE , Campos-Barros A, Toren A, Rozenfeld-Granot G, Carlsson LE, Savige J, . Nonmuscle myosin heavy chain IIA mutations define a spectrum of autosomal dominant macrothrombocytopenias: may-Hegglin anomaly and Fechtner, Sebastian, Epstein, and Alport-like syndromes. Am J Hum Genet 2001;69:1033–45. 11590545 10.1086/324267 PMC1274350

[14] Tzur S , Rosset S, Shemer R, Yudkovsky G, Selig S, Tarekegn A, . Missense mutations in the APOL1 gene are highly associated with end stage kidney disease risk previously attributed to the MYH9 gene. Hum Genet 2010;128:345–50. 20635188 10.1007/s00439-010-0861-0 PMC2921485

[15] Otterpohl KL , Hart RG, Evans C, Surendran K, Chandrasekar I. Nonmuscle myosin 2 proteins encoded by Myh9, Myh10, and Myh14 are uniquely distributed in the tubular segments of murine kidney. Physiol Rep 2017;5:e13513. 29208685 10.14814/phy2.13513 PMC5727274

[16] Ma X , Jana SS, Conti MA, Kawamoto S, Claycomb WC, Adelstein RS. Ablation of nonmuscle myosin II-B and II-C reveals a role for nonmuscle myosin II in cardiac myocyte karyokinesis. Mol Biol Cell 2010;21:3952–62. 20861308 10.1091/mbc.E10-04-0293 PMC2982113

[17] Donaudy F , Snoeckx R, Pfister M, Zenner HP, Blin N, Di Stazio M, . Nonmuscle myosin heavy-chain gene MYH14 is expressed in cochlea and mutated in patients affected by autosomal dominant hearing impairment (DFNA4). Am J Hum Genet 2004;74:770–6. 15015131 10.1086/383285 PMC1181955

[18] Tuzovic L , Yu L, Zeng W, Li X, Lu H, Lu HM, . A human de novo mutation in MYH10 phenocopies the loss of function mutation in mice. Rare Dis 2013;1:e26144. 25003005 10.4161/rdis.26144 PMC3927488

[19] Chandrasekar I , Huettner JE, Turney SG, Bridgman PC. Myosin II regulates activity dependent compensatory endocytosis at central synapses. J Neurosci 2013;33:16131–45. 24107946 10.1523/JNEUROSCI.2229-13.2013 PMC3792455

[20] Chandrasekar I , Goeckeler ZM, Turney SG, Wang P, Wysolmerski RB, Adelstein RS, . Nonmuscle myosin II is a critical regulator of clathrin-mediated endocytosis. Traffic 2014;15:418–32. 24443954 10.1111/tra.12152 PMC3975594

[21] Fernandez K , Wafa T, Fitzgerald TS, Cunningham LL. An optimized, clinically relevant mouse model of cisplatin-induced ototoxicity. Hear Res 2019;375:66–74. 30827780 10.1016/j.heares.2019.02.006 PMC6416072

[22] Benkafadar N , Menardo J, Bourien J, Nouvian R, François F, Decaudin D, . Reversible p53 inhibition prevents cisplatin ototoxicity without blocking chemotherapeutic efficacy. EMBO Mol Med 2017;9:7–26. 27794029 10.15252/emmm.201606230 PMC5210089

[23] Breglio AM , Rusheen AE, Shide ED, Fernandez KA, Spielbauer KK, McLachlin KM, . Cisplatin is retained in the cochlea indefinitely following chemotherapy. Nat Commun 2017;8:1654. 29162831 10.1038/s41467-017-01837-1 PMC5698400

[24] Santos N , Ferreira RS, Santos ACD. Overview of cisplatin-induced neurotoxicity and ototoxicity, and the protective agents. Food Chem Toxicol 2020;136:111079. 31891754 10.1016/j.fct.2019.111079

[25] Burkovics P , Hajdú I, Szukacsov V, Unk I, Haracska L. Role of PCNA-dependent stimulation of 3'-phosphodiesterase and 3'-5' exonuclease activities of human Ape2 in repair of oxidative DNA damage. Nucleic Acids Res 2009;37:4247–55. 19443450 10.1093/nar/gkp357 PMC2715233

[26] Lin Y , Bai L, Cupello S, Hossain MA, Deem B, McLeod M, . APE2 promotes DNA damage response pathway from a single-strand break. Nucleic Acids Res 2018;46:2479–94. 29361157 10.1093/nar/gky020 PMC5861430

[27] Fang J , Zhang WC, Yamashita T, Gao J, Zhu MS, Zuo J. Outer hair cell-specific prestin-CreERT2 knockin mouse lines. Genesis 2012;50:124–31. 21954035 10.1002/dvg.20810 PMC3261330

[28] Ventura A , Kirsch DG, McLaughlin ME, Tuveson DA, Grimm J, Lintault L, . Restoration of p53 function leads to tumour regression in vivo. Nature 2007;445:661–5. 17251932 10.1038/nature05541

[29] Pecci A , Klersy C, Gresele P, Lee KJ, De Rocco D, Bozzi V, . MYH9-related disease: a novel prognostic model to predict the clinical evolution of the disease based on genotype-phenotype correlations. Hum Mutat 2014;35:236–47. 24186861 10.1002/humu.22476 PMC6233870

[30] Li Y , Zeng S, Zhou F, Jie H, Yu D, Hou S, . Overexpression of XIAP inhibits cisplatin-induced hair cell loss. Biochim Biophys Acta Mol Cell Res 2022;1869:119204. 35026350 10.1016/j.bbamcr.2021.119204

[31] Pecci A , Ma X, Savoia A, Adelstein RS. MYH9: structure, functions and role of non-muscle myosin IIA in human disease. Gene 2018;664:152–67. 29679756 10.1016/j.gene.2018.04.048 PMC5970098

[32] Lalwani AK , Atkin G, Li Y, Lee JY, Hillman DE, Mhatre AN. Localization in stereocilia, plasma membrane, and mitochondria suggests diverse roles for NMHC-IIa within cochlear hair cells. Brain Res 2008;1197:13–22. 18241845 10.1016/j.brainres.2007.12.058 PMC2757014

[33] Xie C , Wang FY, Sang Y, Chen B, Huang JH, He FJ, . Mitochondrial micropeptide STMP1 enhances mitochondrial fission to promote tumor metastasis. Cancer Res 2022;82:2431–43. 35544764 10.1158/0008-5472.CAN-21-3910

[34] Reyes A , He J, Mao CC, Bailey LJ, Di Re M, Sembongi H, . Actin and myosin contribute to mammalian mitochondrial DNA maintenance. Nucleic Acids Res 2011;39:5098–108. 21398640 10.1093/nar/gkr052 PMC3130256

[35] Willis J , Patel Y, Lentz BL, Yan S. APE2 is required for ATR-Chk1 checkpoint activation in response to oxidative stress. Proc Natl Acad Sci U S A 2013;110:10592–7. 23754435 10.1073/pnas.1301445110 PMC3696815

[36] Meek DW , Anderson CW. Posttranslational modification of p53: cooperative integrators of function. Cold Spring Harb Perspect Biol 2009;1:a000950. 20457558 10.1101/cshperspect.a000950 PMC2882125

[37] Guikema JE , Linehan EK, Esa N, Tsuchimoto D, Nakabeppu Y, Woodland RT, . Apurinic/apyrimidinic endonuclease 2 regulates the expansion of germinal centers by protecting against activation-induced cytidine deaminase-independent DNA damage in B cells. J Immunol 2014;193:931–9. 24935922 10.4049/jimmunol.1400002 PMC4105697

[38] Guikema JE , Gerstein RM, Linehan EK, Cloherty EK, Evan-Browning E, Tsuchimoto D, . Apurinic/apyrimidinic endonuclease 2 is necessary for normal B cell development and recovery of lymphoid progenitors after chemotherapeutic challenge. J Immunol 2011;186:1943–50. 21228350 10.4049/jimmunol.1002422 PMC4041036

[39] Mihara M , Erster S, Zaika A, Petrenko O, Chittenden T, Pancoska P, . p53 has a direct apoptogenic role at the mitochondria. Mol Cell 2003;11:577–90. 12667443 10.1016/s1097-2765(03)00050-9

[40] Leu JI , Dumont P, Hafey M, Murphy ME, George DL. Mitochondrial p53 activates Bak and causes disruption of a Bak-Mcl1 complex. Nat Cell Biol 2004;6:443–50. 15077116 10.1038/ncb1123

[41] Chipuk JE , Kuwana T, Bouchier-Hayes L, Droin NM, Newmeyer DD, Schuler M, . Direct activation of Bax by p53 mediates mitochondrial membrane permeabilization and apoptosis. Science 2004;303:1010–4. 14963330 10.1126/science.1092734

[42] Freyer DR , Brock PR, Chang KW, Dupuis LL, Epelman S, Knight K, . Prevention of cisplatin-induced ototoxicity in children and adolescents with cancer: a clinical practice guideline. Lancet Child Adolesc Health 2020;4:141–50. 31866182 10.1016/S2352-4642(19)30336-0 PMC7521149

[43] Dhillon S . Sodium thiosulfate: pediatric first approval. Paediatr Drugs 2023;25:239–44. 36517667 10.1007/s40272-022-00550-x

[44] Wheate NJ , Walker S, Craig GE, Oun R. The status of platinum anticancer drugs in the clinic and in clinical trials. Dalton Trans 2010;39:8113–27. 20593091 10.1039/c0dt00292e

[45] Umugire A , Nam YS, Nam YE, Choi YM, Choi SM, Lee S, . Protective effect of avenanthramide-C on auditory hair cells against oxidative stress, inflammatory cytokines, and DNA damage in cisplatin-induced ototoxicity. Int J Mol Sci 2023;24:2947. 36769271 10.3390/ijms24032947 PMC9918115

[46] Kim HJ , Pandit A, Oh GS, Shen A, Lee SB, Khadka D, . Dunnione ameliorates cisplatin ototoxicity through modulation of NAD(+) metabolism. Hear Res 2016;333:235–46. 26341473 10.1016/j.heares.2015.08.017

[47] Gu J , Wang X, Chen Y, Xu K, Yu D, Wu H. An enhanced antioxidant strategy of astaxanthin encapsulated in ROS-responsive nanoparticles for combating cisplatin-induced ototoxicity. J Nanobiotechnology 2022;20:268. 35689218 10.1186/s12951-022-01485-8 PMC9185887

[48] Tan X , Zhou Y, Agarwal A, Lim M, Xu Y, Zhu Y, . Systemic application of honokiol prevents cisplatin ototoxicity without compromising its antitumor effect. Am J Cancer Res 2020;10:4416–34. 33415008 PMC7783741

[49] Kim YR , Do JM, Kim KH, Stoica AR, Jo SW, Kim UK, . C-Phycocyanin from limnothrix species KNUA002 alleviates cisplatin-induced ototoxicity by blocking the mitochondrial apoptotic pathway in auditory cells. Mar Drugs 2019;17:235. 31010222 10.3390/md17040235 PMC6521143

[50] Wang N , Xu A, Zhang H, Zhang Y, Sui R, Fan X, . Melatonin attenuates cisplatin-induced ototoxicity via regulating the cell apoptosis of the inner ear. Comput Math Methods Med 2022;2022:7160816. 36092781 10.1155/2022/7160816 PMC9458396

[51] Lu W , Ni K, Li Z, Xiao L, Li Y, Jiang Y, . Salubrinal protects against cisplatin-induced cochlear hair cell endoplasmic reticulum stress by regulating eukaryotic translation initiation factor 2α signalling. Front Mol Neurosci 2022;15:916458. 35706425 10.3389/fnmol.2022.916458 PMC9189388

[52] Xu B , Li J, Chen X, Kou M. Puerarin attenuates cisplatin-induced apoptosis of hair cells through the mitochondrial apoptotic pathway. Biochim Biophys Acta Mol Cell Res 2022;1869:119208. 35032475 10.1016/j.bbamcr.2021.119208

[53] Yin H , Zhang H, Kong Y, Wang C, Guo Y, Gao Y, . Apelin protects auditory cells from cisplatin-induced toxicity in vitro by inhibiting ROS and apoptosis. Neurosci Lett 2020;728:134948. 32278025 10.1016/j.neulet.2020.134948

[54] Kim J , Cho HJ, Sagong B, Kim SJ, Lee JT, So HS, . Alpha-lipoic acid protects against cisplatin-induced ototoxicity via the regulation of MAPKs and proinflammatory cytokines. Biochem Biophys Res Commun 2014;449:183–9. 24796665 10.1016/j.bbrc.2014.04.118

[55] Hossain MA , Lin Y, Driscoll G, Li J, McMahon A, Matos J, . APE2 is a general regulator of the ATR-Chk1 DNA damage response pathway to maintain genome integrity in pancreatic cancer cells. Front Cell Dev Biol 2021;9:738502. 34796173 10.3389/fcell.2021.738502 PMC8593216

