# Supplementary figures and images for: Cisplatin-Induced APE2 Overexpression Disrupts MYH9 Function and Causes Hearing Loss
Source: Cancer Res Commun. 2025 Jun 20;5(6):994–1007. doi: 10.1158/2767-9764.CRC-24-0506 (PMC12179588; doi:10.1158/2767-9764.CRC-24-0506)

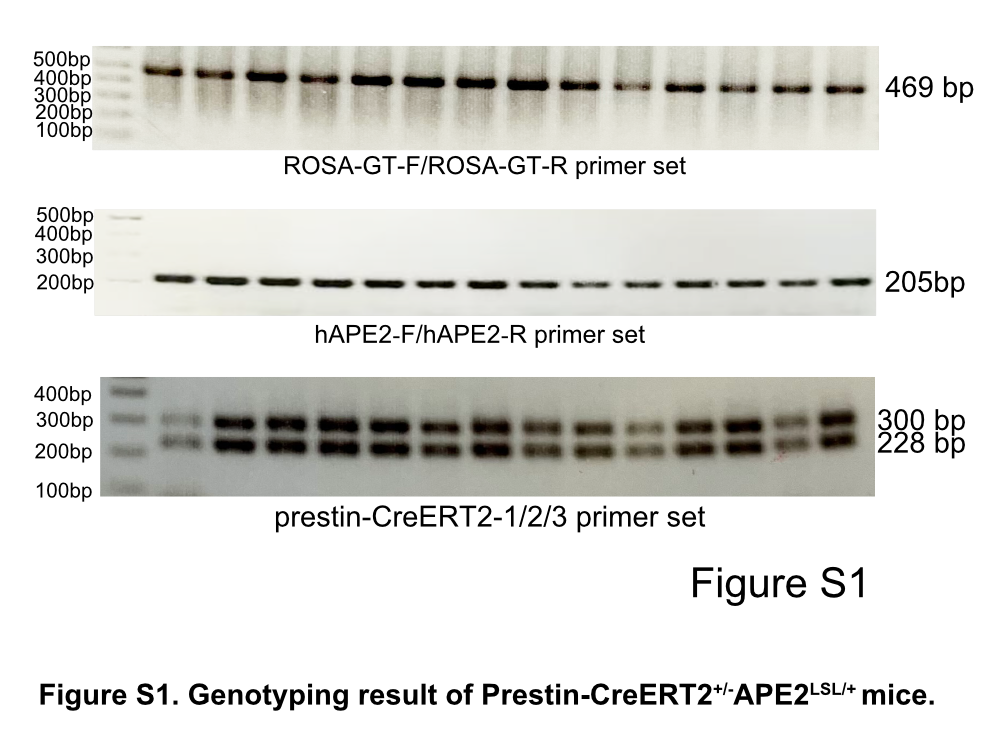

Supplement: Figure S1 [file crc-24-0506_figure_s1_suppsf1.png]

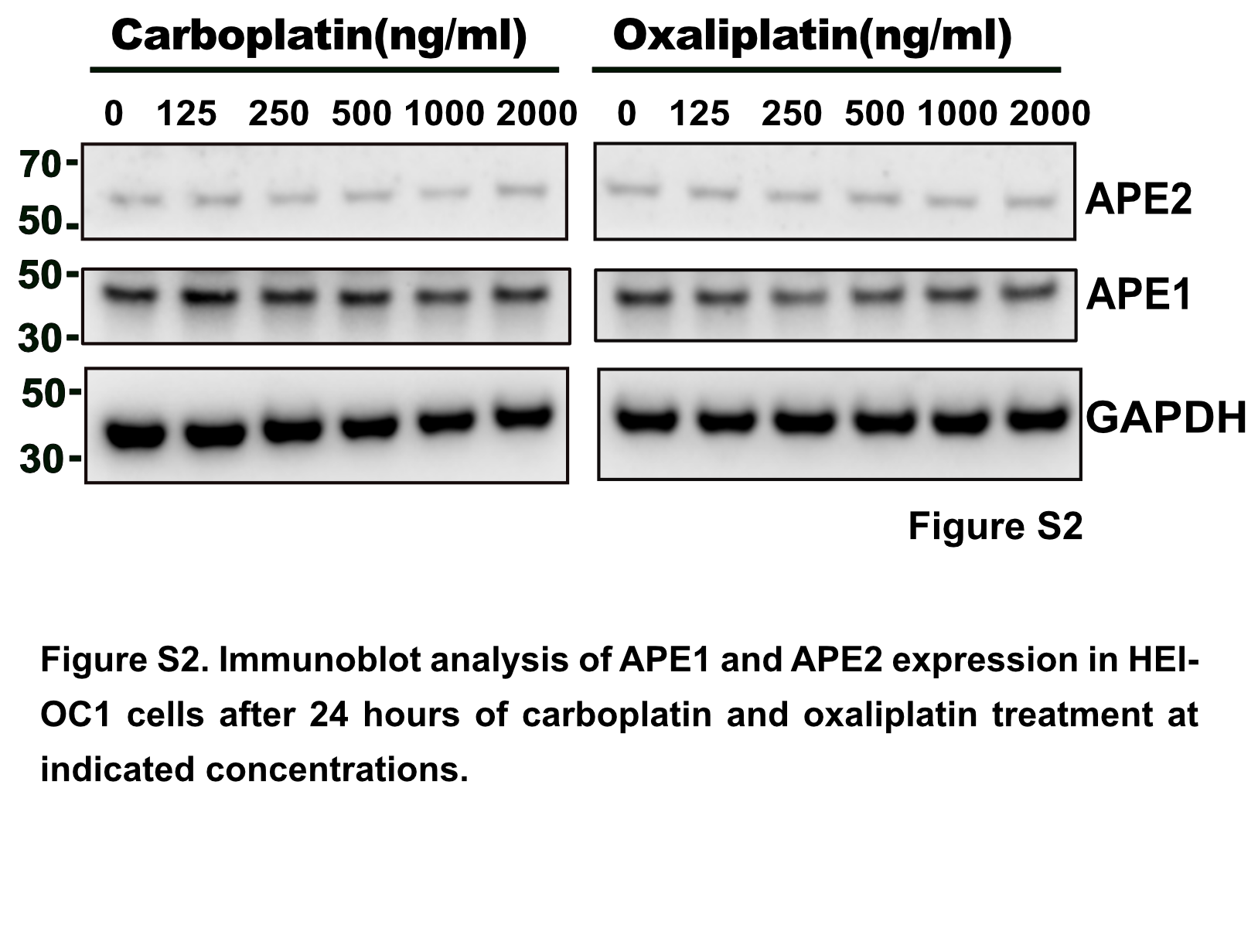

Supplement: Figure S2 [file crc-24-0506_figure_s2_suppsf2.png]

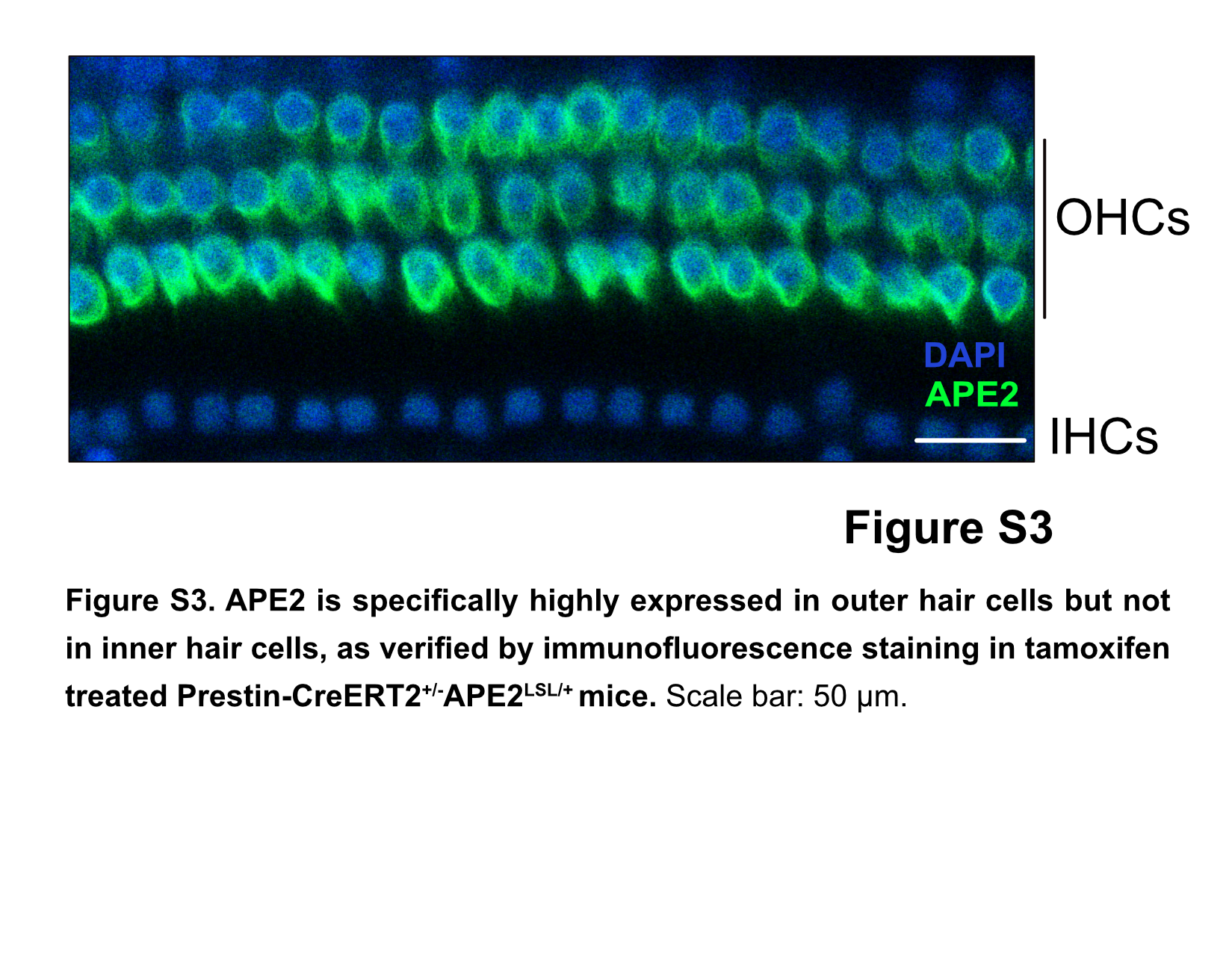

Supplement: Figure S3 [file crc-24-0506_figure_s3_suppsf3.png]

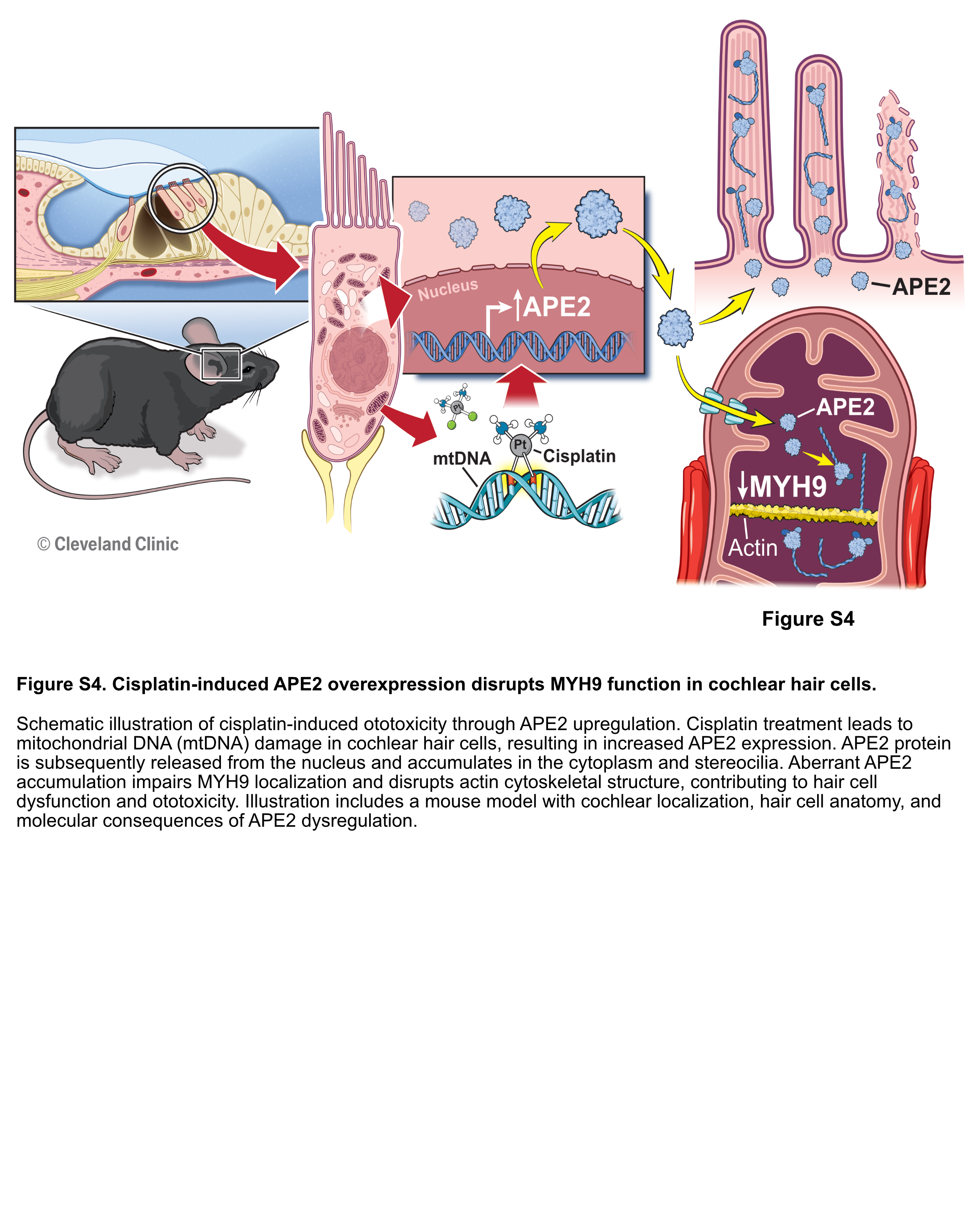

Supplement: Figure S4 [file crc-24-0506_figure_s4_suppsf4.png]
