# Supplementary material for: Cisplatin-Induced APE2 Overexpression Disrupts MYH9 Function and Causes Hearing Loss
Source: Cancer Res Commun. 2025 Jun 20;5(6):994–1007. doi: 10.1158/2767-9764.CRC-24-0506 (PMC12179588; doi:10.1158/2767-9764.CRC-24-0506)
Supplement: Supplementary methods [file crc-24-0506_supplementary_methods_suppsm.docx]

**Supplementary materials and methods**

**Mice**

A CRISPR-mediated recombination Rosa26 3 ’UTR locus was used to generate human APE2 conditional transgenic mice termed hAPE2^LSL^ mice. hAPE2^LSL^ mice were generated by inserting a CAG promoter-driven plasmid with 5X STOP cassette flanked by LoxP sites (LSL) at the start of the hAPE2 cDNAs into mouse Rosa26 3 ’UTR locus. The STOP cassette prevents expression of the hAPE2 gene. Genotyping of hAPE2^LSL^ mice was performed using the following primers:: ROSA-GT-F:5’-AGTCGCTCTGAGTTGTTATCAG-3’; ROSA-GT-R: 5’-TGAGCATGTCTTTAATCTACCTCGATG-3’; hAPE2-F: 5’-GCTGATCCGGAACCCTTAA-3’; hAPE2-R:5’-TTGCTGGGTTCCTGATTTGC-3’. PCR amplification of the ROSA-GT-F/R primer set produces a 469 bp band, confirming the presence of the wild type allele. The hAPE2-F/R primer set yields a 205 bp band, verifying the expression of the hAPE2^LSL^ transgene allele (Fig. S1). To remove the STOP cassette and activate hAPE2 expression in outer hair cells *in vivo*, the hAPE2^LSL^ mice were mated with Prestin-Cre-Estrogen Receptor-T2 (Prestin-CreERT2^+/-^) mice(1) (Kindly provided by Dr. Jian Zuo, Creigh University), in which the ERT moiety retains Cre recombinase activity in the cytoplasm of outer hair cells until tamoxifen administration, which releases this block and promotes recombination of genomic LoxP sites. The genotyping primers for Prestin-CreERT2^+/-^ mice are: prestin-CreERT2-1: CACAAGTTGTGAATGACCTC; prestin-CreERT2-2: GTTAAAGAGCGTAATCTGGAACA; prestin-CreERT2-3: TAACTGCTAGCATTTCCCTT. PCR amplification produces two bands in Prestin-CreERT2^+/-^  mice: a 300 bp band for the wild-type allele and a 228 bp band for the Prestin-CreERT2 allele (Fig. S1). Three-week-old Prestin-CreERT2^+/-^APE2^LSL/+^ mice received intraperitoneal injections of 10 mg/kg of tamoxifen daily for three consecutive days. Prestin-CreERT2^+/-^APE2^LSL/+^ sibling mice without tamoxifen injection were used as control. Both male and female mice were included in the study and randomly assigned to control or experimental groups.

**Gapmer APE2 ASO flanked by blocks of 2’-O modified DNAs**

ASO were synthesized by Integrated DNA Technologies (IDT) or Eurofins Genomics. The sequence of APE2 was: 5’-mU*mG*mU* C*C*T* T*A*C* A*G*A* A*mG*mG* mU- 3’.

The scrambled sequence (GFP ASO as negative control) for 2’-O-Me modified DNA was:

(5’- mA*mA*mG*mG*mC*A*A*G*C*U*G*A*C*C*C*mU*mG*mA*mA*mG - 3’. m= 2’OMe; ‘*’ = phosphorothioate (PS) linkage

**Plasmids**

PMEV-3FLAG-hAPE2 plasmid was constructed by using hAPE2 cDNA cloned into PMEV-3FLAG-empty vector. 15 MYH9 EGFP fusion protein (including full length and different fragments) expression plasmids was constructed for transfection with PMEV-3FLAG-hAPE2 plasmid to identify the APE2 and MYH9 binding site. APE2 colocalization with 15 footprint constructs of MYH9 fragment fusion protein with GFP plasmid were analyzed using Volocity software (Quorum Technologies Inc, RRID:SCR_002668).
